# Enhanced Protective Efficacy of Nonpathogenic Recombinant *Leishmania tarentolae* Expressing Cysteine Proteinases Combined with a Sand Fly Salivary Antigen

**DOI:** 10.1371/journal.pntd.0002751

**Published:** 2014-03-27

**Authors:** Farnaz Zahedifard, Elham Gholami, Tahereh Taheri, Yasaman Taslimi, Fatemeh Doustdari, Negar Seyed, Fatemeh Torkashvand, Claudio Meneses, Barbara Papadopoulou, Shaden Kamhawi, Jesus G. Valenzuela, Sima Rafati

**Affiliations:** 1 Molecular Immunology and Vaccine Research Laboratory, Pasteur Institute of Iran, Tehran, Iran; 2 Biotechnology Research Center, Pasteur Institute of Iran, Tehran, Iran; 3 Vector Molecular Biology Section, Laboratory of Malaria and Vector Research, National Institute of Allergy and Infectious Diseases, National Institute of Health, Rockville, Maryland, United States of America; 4 Research Centre in Infectious Disease, CHUL Research Centre (CHU de Québec Research Centre) and Department of Microbiology, Infectious Disease and Immunology, Laval University, Quebec, Canada; Yale School of Public Health, United States of America

## Abstract

**Background:**

Novel vaccination approaches are needed to prevent leishmaniasis. Live attenuated vaccines are the gold standard for protection against intracellular pathogens such as *Leishmania* and there have been new developments in this field. The nonpathogenic to humans lizard protozoan parasite, *Leishmania* (*L*) *tarentolae*, has been used effectively as a vaccine platform against visceral leishmaniasis in experimental animal models. Correspondingly, pre-exposure to sand fly saliva or immunization with a salivary protein has been shown to protect mice against cutaneous leishmaniasis.

**Methodology/Principal Findings:**

Here, we tested the efficacy of a novel combination of established protective parasite antigens expressed by *L. tarentolae* together with a sand fly salivary antigen as a vaccine strategy against *L. major* infection. The immunogenicity and protective efficacy of different DNA/Live and Live/Live prime-boost vaccination modalities with live recombinant *L. tarentolae* stably expressing cysteine proteinases (type I and II, CPA/CPB) and PpSP15, an immunogenic salivary protein from *Phlebotomus papatasi*, a natural vector of *L. major*, were tested both in susceptible BALB/c and resistant C57BL/6 mice. Both humoral and cellular immune responses were assessed before challenge and at 3 and 10 weeks after *Leishmania* infection. In both strains of mice, the strongest protective effect was observed when priming with PpSP15 DNA and boosting with PpSP15 DNA and live recombinant *L. tarentolae* stably expressing cysteine proteinase genes.

**Conclusion/Significance:**

The present study is the first to use a combination of recombinant *L. tarentolae* with a sand fly salivary antigen (PpSP15) and represents a novel promising vaccination approach against leishmaniasis.

## Author Summary

More than 98 countries are reported as endemic for leishmaniasis, a vector-borne disease transmitted by sand flies. Drug-resistant forms have emerged and there is an increased need to develop advanced preventive strategies. Live attenuated vaccines are the gold standard for protection against intracellular pathogens such as *Leishmania* and there have been new developments in this field. The lizard protozoan parasite, *L. tarentolae*, is nonpathogenic to humans and has been used effectively as a vaccine platform against visceral leishmaniasis in experimental animal models. Correspondingly, pre-exposure to sand fly saliva or immunization with salivary proteins has been shown to protect mice against cutaneous leishmaniasis. Herein, we used DNA/Live and Live/Live prime-boost vaccination strategies against cutaneous leishmaniasis based on recombinant *L. tarentolae* stably expressing *CPA/CPB* genes with and without the sand fly salivary antigen PpSP15 in both resistant and susceptible mice models. Assessment of the immune response and parasite burden in vaccinated mice at different time intervals post-challenge demonstrated that combination of recombinant *L. tarentolae CPA/CPB* with PpSP15 DNA elicits an enhanced protective immune response against cutaneous leishmaniasis in mice. This parasite- and insect vector-derived antigen combination represents an important step forward in the development of new vaccine strategies against *Leishmania* infections.

## Introduction

Leishmaniasis is one of the greatest health challenges in nearly 98 countries, contributing to 2 million new clinical cases per year in tropical and subtropical regions of the globe [1]. The disease is transmitted by sandflies and is manifested in several clinical forms, mainly cutaneous leishmaniasis (CL), mucocutaneous leishmaniasis (MCL), and visceral leishmaniasis (VL) [2]. The geographical spread of the various clinical forms depends on vector availability. For instance, over 90% of CL cases occur in Afghanistan, Algeria, Brazil, Iran, Peru, Saudi Arabia and Syria; while, 95% of VL cases are found in Bangladesh, India, Nepal, Sudan, Ethiopia and Brazil [3]. High treatment costs, toxicity of drugs, and the constant emergence of parasite resistance highlight the need for a vaccine. Despite the observation that individuals with a healed primary *Leishmania* infection are protected against reinfection, no effective vaccine has been developed thus far. Lack of success may be due to our incomplete understanding of the control and regulation of immune responses during infection/reinfection and the mechanisms involved in the development of immune memory. In humans, acquired resistance to *L. major* infection is mediated primarily by cellular immunity, in particular antigen-specific type 1 T helper (Th1) cells. Similarly, Th1 dependent protection is observed in mouse experimental models of *L. major* infection. Most efforts for antigen identification have been focused on parasite proteins. More recently, it was shown that immunization with defined sand fly salivary proteins confers protection against leishmaniasis [4]. This suggests that salivary molecules can contribute to protection as a component of an anti-*Leishmania* vaccine.

Live attenuated vaccines are the gold standard for protection against intracellular pathogens. Importantly, there have been some recent attempts using this approach for the development of *Leishmania* vaccines [5], [6]. Other approaches manipulate the *Leishmania* genome to engineer genetically modified parasites by introducing or eliminating particular virulence genes [7], [8], [9]. These approaches are powerful alternatives for the development of new generation vaccines against leishmaniasis. Nonpathogenic to humans *Leishmania* strains are also being assessed as promising vaccine tools [10]. Vaccination with a *L. tarentolae* recombinant strain expressing select immunogenic components of *L. infantum*, including the *A2* and the cysteine proteinases A and B (*CPA/CPB*) genes as a tri-fusion conferred protection against *L. infantum* infection [11].

In the present study, we evaluated the efficacy of a new prime-boost vaccine combination consisting of a live recombinant nonpathogenic parasite and a vector salivary protein in eliciting a more powerful protective immunity against *L. major* infection. For this, we combined a recombinant *L. tarentolae* expressing the *CPA/CPB* cysteine proteinases with the immunogenic sand fly salivary molecule PpSP15 delivered as a DNA vaccine. We used different prime-boost regimens and evaluated the immunogenicity and protective effectiveness of this novel vaccine combination against *L. major* infection in both BALB/c and C57BL/6 mice.

## Materials and Methods

### Ethics statement

All mouse experiments including maintenance, animals' handling program and blood sample collection were approved by Institutional Animal Care and Research Advisory Committee of Pasteur Institute of Iran (Research deputy dated October 2010), based on the Specific National Ethical Guidelines for Biochemical Research issued by the Research and Technology Deputy of Ministry of Health and Medicinal Education (MOHM) of Iran that was issued in 2005.

### Reagents

All solutions were prepared using MilliQ ultrapure (Milli-QSystem, Millipore, Molsheim, France) and non-pyrogenic water to avoid surface-active impurities. G418, and Sodium dodecyl sulfate (SDS) were purchased from Sigma-Aldrich (Sigma, Deisenhofen, Germany). The material for PCR, enzymatic digestion and agarose gel electrophoresis were acquired from Roche Applied Sciences (Mannheim, Germany). Cell culture reagents including M199 medium, HEPES, L-glutamine, adenosine, hemin, gentamicin, DMEM and Schneider were purchased from Sigma (Darmstadt, Germany) and Gibco (Gibco, Life Technologies GmbH, Karlsruhe, Germany), respectively. Fetal Calf Sera (FCS) was purchased from Gibco (Gibco, Life Technologies GmbH, Karlsruhe, Germany). All cytokine kits were purchased from DuoSet R & D kits, (Minneapolis, USA).

### DNA constructs

A 2.3 kb fragment content *CPA/CPB/EGFP* fused genes (with stop codons at the end of the *EGFP* ORF) was digested from pCB6-*CPA/CPB/EGFP* using *Sac*I and *BamH*I and then cloned into the corresponding sites of pEGFP-N1 vector (Clontech, Palo Alto, CA) to provide the vector referred to as pEGFP-*CPA/CPB/EGFP*. After confirmation of the tri-fused gene through PCR and sequence determination, the pLEXSY-NEO2 vector (EGE-233, Jena bioscience, Germany) was used as an integrative vector to incorporate the *CPA/CPB/EGFP* fusion gene into the genome of the parasite. The *CPA/CPB/EGFP* was digested from pEGFP-*CPA/CPB/EGFP* using *Xho*I and *Xba*I and cloned into *Nhe*I and *Xho*I sites of the pLEXSY vector (*Xba*I and *Nhe*I are isoschizomers and make compatible sticky ends). For integration, the *Swa*I was used to linearize the vector at the 5′ and 3′ ends. Then the *L. tarentolae* (Tar II ATCC 30267) was grown in M199 5% inactivated fetal bovine serum (iFBS) to an optimal concentration. Parasite density was measured by counting the cells dissolved in Hyman's solution (HgCl_2_ 0.5 g, NaCl 1 g, Na_2_SO_4_, 10H_2_O 11.5 g) using a hemocytometer. The pellet was resuspended in ice-cold electroporation buffer (21 mM HEPES, 137 Mm NaCl, 5 mM KCl, 0.7 mM Na_2_HPO_4_, 6 mM glucose; pH 7.5) to a final density of 10^8^ parasites/ml, as recommended [12]. A total of 4.0×10^7^ parasites/300 µl were mixed with 5–10 µg linearized DNA for stable transfection in a 0.2 cm electroporation cuvette (BioRad, USA) and stored on ice for 10 min. Electroporations were performed twice at 450 V, 500 µF using Bio Rad Gene Pulser Ecell device (Bio-Rad, USA) and the cell suspension was immediately put on ice for 10 min. Electroporated parasites were then transferred to 3 ml complete M199 media supplemented with 10% iFBS free of antibiotic and incubated at 26°C for 24 hours. Then, the live parasites were collected by centrifugation at 3000 rpm for 10 min at 4°C. Cells were subsequently transferred onto semi-solid plates of M199 medium containing 50% Noble agar (Difco, USA) and 50 µg/ml G418 (Gibco, Germany) and incubated at 26°C. The genotype of transfected parasites was confirmed by Southern blotting using the *EGFP*ORF through incorporation of radiolabeled dCTP in a PCR reaction. In addition, genomic DNA obtained from transfected and wild type (WT) cells was amplified by PCR with specific primers to the upstream and downstream of the flanking region of 5′SSU. Forward primer (F3001) anneals upstream of the 5′SSU on WT genome and reverse primer (A1715) anneals to the backbone of the vector, downstream of 5′SSU and upstream of *CPA/CPB/EGFP* gene. The sequences for primer F3001 are: 5′ GAT CTG GTT GAT TCT GCC AGT AG 3′ and for primer A1715: 5′ TAT TCG TTG TCA GAT GGC GCA C 3′. The expression of CPA/CPB in the recombinant parasites was confirmed by RT-PCR, Western blot as well as flow cytometry.

### DNA vaccine constructs

The gene coding for PpSP15 (NCBI accession number: AF335487) from the NH2 terminus to the stop codon was amplified from *P. papatasi* SP15-specific cDNA by PCR as reported previously [13] and cloned into the TOPO TA cloning vector PCRII (Invitrogen). The plasmid VR1020-SP15 was purified using the Endo Free Plasmid Mega kit according to the manufacturer's instructions (QIAGEN, Germany).

### Antigens and salivary gland preparation

Frozen and thawed (F/T) *L. major* and *L. tarentolae CPA/CPB/EGFP* antigens were prepared from stationary phase promastigotes. Parasites were washed with PBS (8 mM Na2HPO4, 1.75 mM KH_2_PO4, 0.25 mM KCl, 137 mM NaCl) prior to 10 times exposition to liquid nitrogen and 37°C water bath alternately. The rCPA and rCPB were also prepared as previously reported [14]. Protein concentrations were measured with a BCA kit (PIERCE, Chemical Co., Rochford III). For preparation of salivary gland homogenate (SGH), *P. papatasi* females, Israeli strain, were used for dissection of salivary glands 3–7 d after emergence as previously described [15]. Briefly, salivary glands were disrupted by ultra-sonication and centrifuged at 10,000 g for 3 min and the resultant supernatant was dried in a Speed Vac (Thermo Scientific) and reconstituted before use in the listed experiments.

### Vaccination regimens and infectious challenge

Female BALB/c (H-2^d^) and C57BL6 (H-2^b^) mice (6–8 weeks old, weighting 20±5 g) were purchased from the Pasteur Institute of Iran animal breeding facilities. All animals were housed in plastic cages with free access to tap water and standard rodent pellets in an air-conditioned room under a constant 12∶12 h light-dark cycle at room temperature and 50–60% relative humidity. Six groups of BALB/c or C57BL/6 mice (n = 20 per group) were vaccinated in different prime/boost modalities given three weeks apart in the right hind footpad (Table 1). These included, G1: vaccination with *L. tarentolae CPA/CPB/EGFP+* and boosting with *L. tarentolae CPA/CPB/EGFP+*; G2: vaccination with VR1020-SP15 and boosting with *L. tarentolae CPA/CPB/EGFP+* followed by VR1020-SP15 the day after; G3: vaccination with *L. tarentolae CPA/CPB/EGFP+* followed by VR1020-SP15 the next day and boosting with *L. tarentolae CPA/CPB/EGFP+* followed by VR1020-SP15 the next day; G4: Control group, vaccination with PBS; G5: vaccination and boosting with VR1020-SP15; G6: vaccination and boosting with *L. tarentolae* EGFP+. *L. major* EGFP^+^ (MRHO/IR/75/ER) parasites were used for the infectious challenge and were kept in a virulent state by continuous passage in BALB/c mice. The promastigotes were cultured in M199 medium supplemented with 5% iFBS and 40 mM HEPES, 0.1 mM adenosine, 0.5 µg/ml hemin, 2 mM L-glutamine and 50 µg/ml gentamicin at 26°C. For mice challenge, a total of 2×10^5^ stationary phase promastigotes were injected subcutaneously in the left hind footpad 3 weeks after the booster immunization. For G2, G3, G4 and G5, 0.5 pair of sand fly SGH was mixed with parasites and used for challenge.

**Table 1:** Vaccination regimens in BALB/c and C57BL/6 mice models.

Groups	Prime	Boost	Challenge	Modality
Group 1 N = 20	*L. tarentolae* (CPA/CPB/EGFP) 2×10^7^	*L. tarentolae* (CPA/CPB/EGFP) 2×10^7^	*L. major* Footpad s.c. 2×10^5^	rLive/rLive vaccine
Group 2 N = 20	VR1020-SP15 20 µg/mice	*L. tarentolae* (CPA/CPB/EGFP) 2×10^7^ VR-SP15 (next day)	*L. major*+SGH Footpad s.c. 2×10^5^	DNA/rLive+DNA vaccine
Group 3 N = 20	*L. tarentolae* (CPA/CPB/EGFP) 2×10^7^ VR1020-SP15 (next day)	*L. tarentolae* (CPA/CPB/EGFP) 2×10^7^ VR1020-SP15 (next day)	*L. major*+SGH Footpad s.c. 2×10^5^	rLive+DNA/rLive+DNA vaccine
Group 4 N = 20	PBS	PBS	*L. major*+SGH Footpad s.c. 2×10^5^	CONTROL
Group 5 N = 20	VR1020-SP15 20 µg/mice	VR1020-SP15 20 µg/mice	*L. major*+SGH Footpad s.c. 2×10^5^	DNA/DNA vaccine
Group 6 N = 20	*L. tarentolae* 2×10^7^	*L. tarentolae* 2×10^7^	*L. major* Footpad s.c. 2×10^5^	Live/Live vaccine CONTROL

### Cytokine assays before and after challenge with *L. major*

The profile of cytokine production in the groups vaccinated with *L. tarentolae* CPA/CPB/EGFP+ (G1) and a combination of *L. tarentolae CPA/CPB/EGFP+* and VR1020-SP15 (G2, and G3) and the PBS-immunized control group G4 in both BALB/c and C57BL/6 mice was measured before challenge and at 3 and 10 weeks post challenge in two independent repeats. Briefly, at each time point, 4 mice from each group were sacrificed. Their spleen was treated with a tissue grinder and red blood cells were lysed for 5 minutes using the ACK lyses buffer (NH_4_Cl 0.15M, KHCO_3_ 1 mM, Na_2_EDTA 0.1 mM). Splenic cells were then washed, put in culture at 3.5×10^6^ cells/ml and exposed to recombinant antigens rCPA (10 µg/ml) and rCPB (10 µg/ml), F/T lysate of *L. major* (15 µg/ml), *L. tarentolae* harboring cysteine proteinase genes of interest (25 µg/ml), and SGH (2pairs/ml). Cell culture supernatants were collected after 24 hours for IL-2 and TNF-α assays and 72 hours later for IFN-γ and IL-4 assays. Cytokine measurements were performed by Sandwich ELISA using the DuoSet R & D kits as per the manufacturer's instructions. The minimum detection limit is 2 pg/ml for mouse IFN-γ and IL-4, 3 pg/ml for IL-2 and 5 pg/ml for TNF-α. All measurements were run in duplicates for two independent experiments. Concanavalin A (Con A; 5 µg/ml) was used in all experiments as a positive control.

### Determination of Ag-specific antibody responses

For the groups vaccinated with *L. tarentolae* CPA/CPB/EGFP+ (G1) and a combination of *L. tarentolae CPA/CPB/EGFP+* and VR1020-SP15 (G2, and G3) and the PBS-immunized control group G4, mice were bled to obtain serum for determination of antibody responses. The serum sample obtained from each mouse was analyzed by ELISA for specific IgG1 and IgG2a isotype responses three weeks after booster immunization (against F/T lysate of *L. tarentolae* CPA/CPB/EGFP+ (10 µg/ml) and SGH (2pair/ml) and at 5 weeks after challenge against F/T lysate of *L. major* (10 µg/ml) and SGH (2pair/ml). Briefly, 96-well plates (Greiner) were coated with each antigen overnight at 4°C. Plates were blocked with 100 µl of 1% BSA in PBS at 37°C for 2 h to prevent nonspecific binding. Sera (1∶100) were added and incubated for 2 h at 37°C. After three washes, goat anti-mouse IgG1-HPR (1∶10,000, Southern Biotech, Canada) or goat anti-mouse IgG2a-HPR (1∶10,000, Southern Biotech, Canada) were added and incubated for 2 h at 37°C. After four washes, plates were incubated for 30 min at 37°C with Peroxidase Substrate System (KPL, ABTS) as substrate. Reactions were stopped with 1% SDS and the absorbance was measured at 405 nm.

### Parasite burden

The parasite load in different groups of BALB/c and C57BL/6 mice (G1, G2, G3, G4, G5 and G6) were determined by the limiting dilution assay at 3 and 10 weeks post challenge [16]. Briefly, at each time point 4 mice from each group were taken randomly, sacrificed and the lymph nodes (LN) were excised and weighed. After homogenizing, 20 different serial dilutions (10^−1^ to 10^−20^) were prepared in Schneider's *Drosophila* medium supplemented with 10% iFBS and gentamicin (0.01%). Diluted cells were cultured in 96 well plates in duplicate and investigated 7 and 14 days later for positive wells. The parasite load was calculated using the following formula: −Log_10_ (last dilution with live parasites/weight of homogenized LN).

### Imaging of infection intensity of EGFP-transfected *L. major* in the footpad of BALB/c mice

To demonstrate the *in vivo* level of infection, the infected footpad (FP) was imaged 10 weeks after challenge with the KODAK Image Station 4000 Digital Imaging System. Briefly, six BALB/c mice from each group (G1, G2, G3, G4, G5 and G6) were treated with a depilatory substance (Nair) to remove hair from their FPs to reduce background auto fluorescence. Afterward mice were temporarily anesthetized intraperitoneally with a mixture of xylazine 2% (7.5 µl), Ketamine 10% (30 µl) and saline solution (260 µl) per mouse and then imaged. Pixel counting and measurement of the lesions were performed using the KODAK molecular image software version 5.3. Measurements were reported as “Net intensity”, a quantitative measurement defined as the number of green pixels in a given area multiplied by the average intensity of each pixel minus the background intensity.

### Statistical analysis

Statistical analysis was performed using Graph-Pad Prism 5.0 for Windows (San Diego, California). Depending on data passing normality tests, ANOVA or Mann-Whitney U tests were computed. *P* values less than 0.05 were considered significant. The specific test employed is indicated in each figure.

## Results

### Generation of a recombinant *L. tarentolae* expressing the *CPA/CPB/EGFP* tri-fusion gene

**Figure 1:**
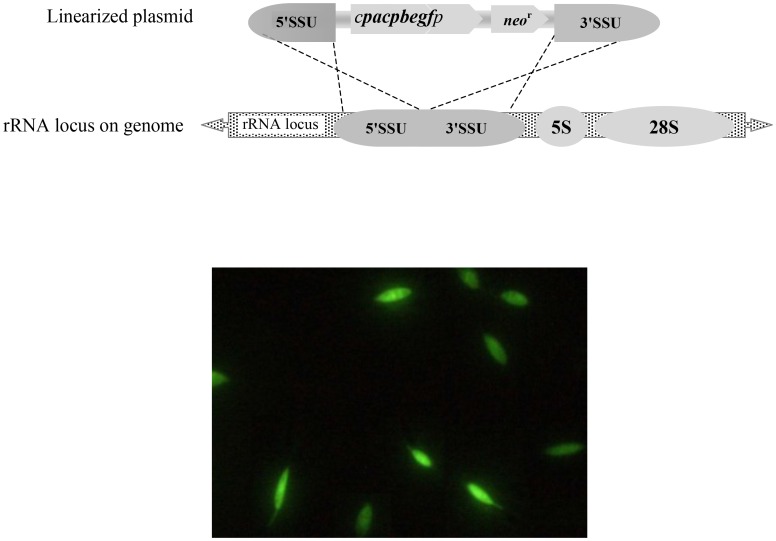
Schematic structure of the 5′SSU-*CPA/CPB/EGFP*-3′SSU cassette linearized with *Swa*I to integrate it into the SSU region of the *L. major* genome (A). Microscopic analysis of *L. tarentolae* stably expressing *CPA/CPB/EGFP* into the ribosomal locus (B).

Expression of the 2.3 kb *CPA/CPB/EGFP* tri-fusion genes in *L. tarentolae* is under the regulatory control of the rRNA Pol I promoter. We integrated the *CPA/CPB/EGFP* fragment flanked by 5′(∼860 bp) and 3′SSU (∼1080 bp) sequences into the rRNA locus of *L. tarentolae* (Figure 1A). The recombinant *L. tarentolae* strain expressing *CPA/CPB/EGFP* genes displayed a normal morphology (a drop-like shape) with a normal length of the flagellum comparable to that of the wild type strain. EGFP expression and intensity were verified by visualization using an epifluorescence microscope. The EGFP was attached to the C-terminal end of CPB and fluorescence is distributed through the whole cytoplasm (Figure 1B). Confirmation of *CPA/CPB/EGFP* expression at the level of RNA and protein was verified using RT-PCR and western blot, respectively (data not shown).

### Pre-challenge evaluation of the immunogenicity of live recombinant *L. tarentolae CPA/CPB/EGFP* combined with PpSP15 in three different regimens in BALB/c and C56BL/6 mice

A major requirement of vaccines is to protect the majority of a population that normally displays a high diversity in MHC haplotypes. It is known that *L. major* causes a non-healing cutaneous infection in susceptible BALB/c mice characterized by progressive skin lesions and visceralization of the parasites to the spleen [17], [18]. In contrast, C57BL/6 mice are naturally resistant against *L. major* and the infection normally causes transient symptoms and is self-healing [18]. Therefore, we evaluated the immune response in both BALB/c (H-2^d^) and C57BL/6 (H-2^b^) mice in the groups vaccinated with *L. tarentolae CPA/CPB/EGFP+* (G1), combination of *L. tarentolae CPA/CPB/EGFP+* and VR1020-SP15 (G2, and G3) and the PBS-immunized control group G4 (Table 1). It has been shown that IFN-γ and TNF-α are important parameters for vaccine evaluation since they synergize their capacity to mediate killing of pathogens. Furthermore, IL-2 also enhances the expansion of T cells, leading to a more efficient effector responses [19]. Since these effector cytokines mediate protection, we evaluated antigen specific immune responses three weeks after booster immunization by measuring the production of IFN-γ, IL-4, IL-2 and TNF-α in the supernatant of splenocytes in response to rCPA/rCPB or F/T lysate of *L. tarentolae CPA/CPB/EGFP*.

**Figure 2:**
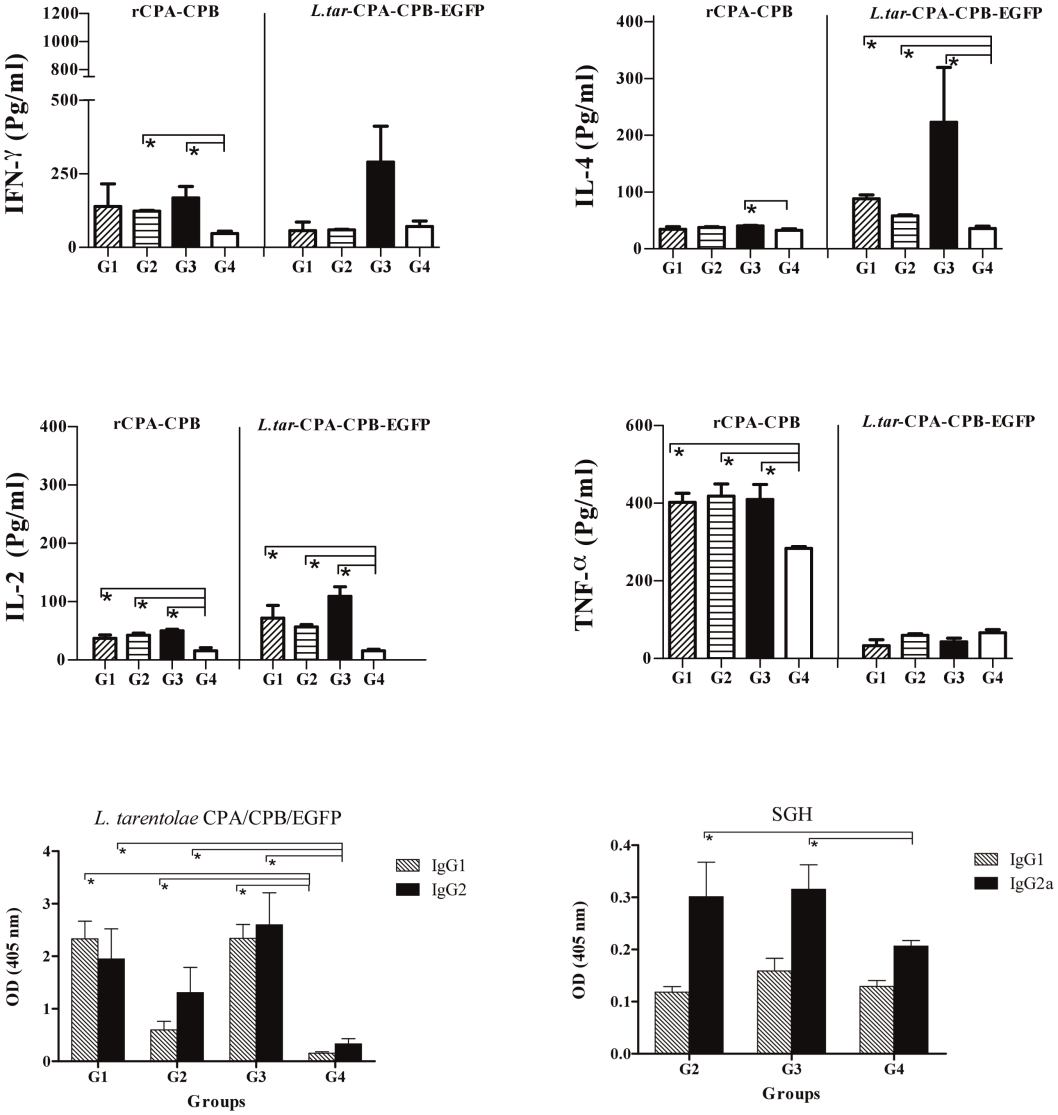
Cellular and humoral immune responses by splenocytes of BALB/c mice after booster immunization. A) Cytokine production from the splenocytes of 4 mice in the designated groups 3 weeks after last immunization. Single cell suspensions were cultured in duplicate in the presence of recombinant CPA/CPB or freeze-thawed recombinant *L. tarentolae*. Culture supernatants were assayed for IFN-γ, IL-4, IL-2 and TNF-α production by ELISA. Each bar represents the Mean±SD of 4 mice per group. B) Antigen specific antibody responses against *L. tarentolae CPA/CPB/EGFP* and SGH before parasite challenge. Data were generated as Mean±SD for sera of individual mice within their respective vaccination group (n = 8–10). The asterisk indicates the significant difference between values at the indicated time points as determined by Mann Whitney U test (*p*<0.05 denoted as *), compared to the control group (G4). Results are representative of two independent experiments.

In susceptible BALB/C mice, the levels of IFN-γ production by splenocytes after rCPA/CPB stimulation were significantly higher (*p<0.05*) in the G2 and G3 vaccinated groups compared to the control-immunized group G4 (Figure 2A). No significant difference in the levels of IFN-γ production was observed in any of the vaccinated groups when stimulation was done with *L. tarentolae CPA/CPB/EGFP* (Figure 2A). We further investigated whether splenocytes from the three different vaccinated regimens secreted the Th2-associated cytokine IL-4. Upon stimulation with rCPA/CPB, G3 exhibited a small but significantly higher level of IL-4 as compared to control group G4 (Figure 2A). Stimulation of splenocytes with *L. tarentolae CPA/CPB/EGFP* resulted in significantly higher levels of IL-4 in G1, G2 andG3 groups as compared to control group G4 (Figure 2A). Furthermore, G1, G2 and G3 produced significantly higher levels of IL-2 compared to G4 (*p*<0.05) when stimulated with rCPA/CPB or *L. tarentolae CPA/CPB/EGFP* (Figure 2A). For TNF-α, G1, G2 and G3 showed significantly higher levels in comparison to control group G4 upon stimulation with rCPA/CPB (Figure 2A) but no difference was observed among these groups after *L. tarentolae CPA/CPB/EGFP* stimulation (Figure 2A).

The antibody response of vaccinated BALB/c mice against *L. tarentolae CPA/CPB/EGFP* for groups G1, G2, G3 and G4, and against sand fly salivary gland homogenate (SGH) for groups G2, G3 and G4 was determined before challenge (Figure 2B). Higher levels of both IgG1 and IG2a antibodies against *L. tarentolae CPA/CPB/EGFP* was observed in vaccinated groups G1, G2 and G3 compared to control group G4 (Figure 2B, *p*<0.05). In both G2 and G3, the level of IgG2a was higher than IgG1 but the opposite was obtained for G1 (Figure 2B, *p*<0.05). When SGH was used as antigen, both the G2 and G3 groups produced significantly higher levels of IgG2a as compared to control group G4 (*p*<0.05) while no significant differences were observed for IgG1 levels in all three vaccinated groups (*p*>0.05).

**Figure 3:**
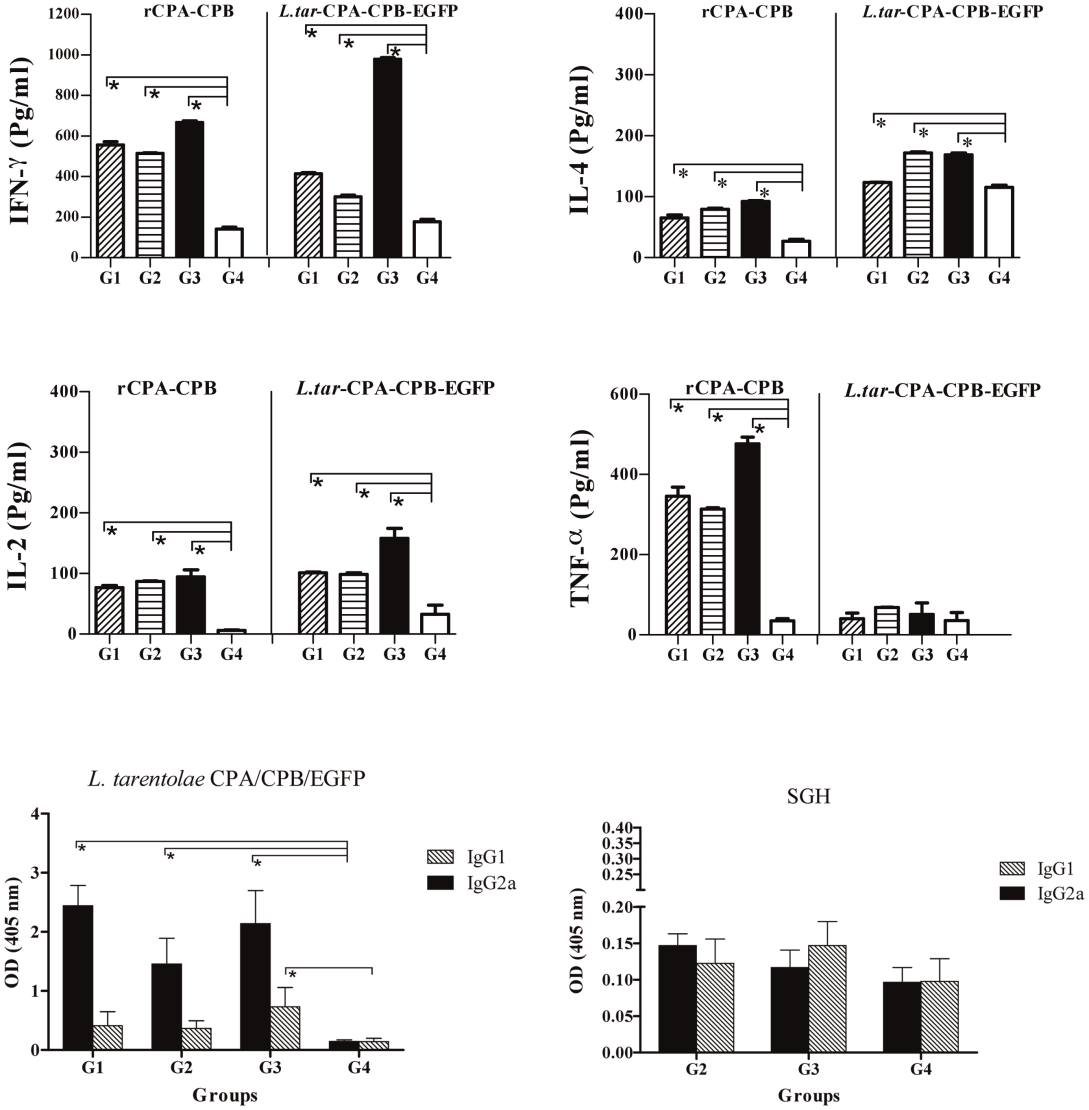
Cellular and humoral immune responses by splenocytes of C57BL/6 mice measured after last immunization. A) Cytokine production from the splenocytes of 4 mice from the different groups at 3 weeks after last immunization. Cells cultured in duplicate in the presence of recombinant CPA/CPB or freeze-thawed recombinant *L. tarentolae*. Culture supernatants were assayed for IFN-γ, IL-4, IL-2 and TNF-α production by ELISA. Each bar represents the Mean±SD of 4 mice per group. B) Antigen specific antibody responses against *L. tarentolae CPA/CPB/EGFP* and SGH before challenge. Data were generated as Mean±SD for sera of individual mice within their respective vaccination group (n = 8–10). The asterisk indicates the significant difference between values at the indicated time points as determined by the Mann Whitney U test (*p*<0.05 denoted as *), compared to the control group (G4). Results are representative of two independent experiments.

For resistant C57BL/6 mice, splenocytes produced significantly higher levels of IFN-γ and IL-4 in the three vaccinated groups (G1, G2, G3) as compared to control group G4 when stimulated with either rCPA/CPB or *L. tarentolae CPA/CPB/EGFP* (Figure 3A, *p*<0.05). Nevertheless, the level of IL-4 was lower than that of IFN-γ in all vaccinated groups (Figure 3A). Groups G1, G2 and G3 produced significantly higher levels of IL-2 compared to G4 (*p*<0.05) when stimulated with rCPA/CPB or *L. tarentolae CPA/CPB/EGFP* (Figure 3A). As for TNF-α, it was only produced upon stimulation with rCPA/CPB where G1, G2 and G3 showed significantly higher levels in comparison to G4 (Figure 3A).

The three vaccinated groups produced significantly higher levels of IgG2a against *L. tarentolae CPA/CPB/EGFP* compared to control G4 (Figure 3B). The level of IgG1 was only significantly higher in G3 as compared to control G4 (p<0.05). Furthermore, there were no significant differences between IgG1 and IgG2a levels against SGH in the three tested groups G2, G3 and G4 (Figure 3B).

### Evaluation of vaccine efficacy in susceptible BALB/c mice

**Figure 4:**
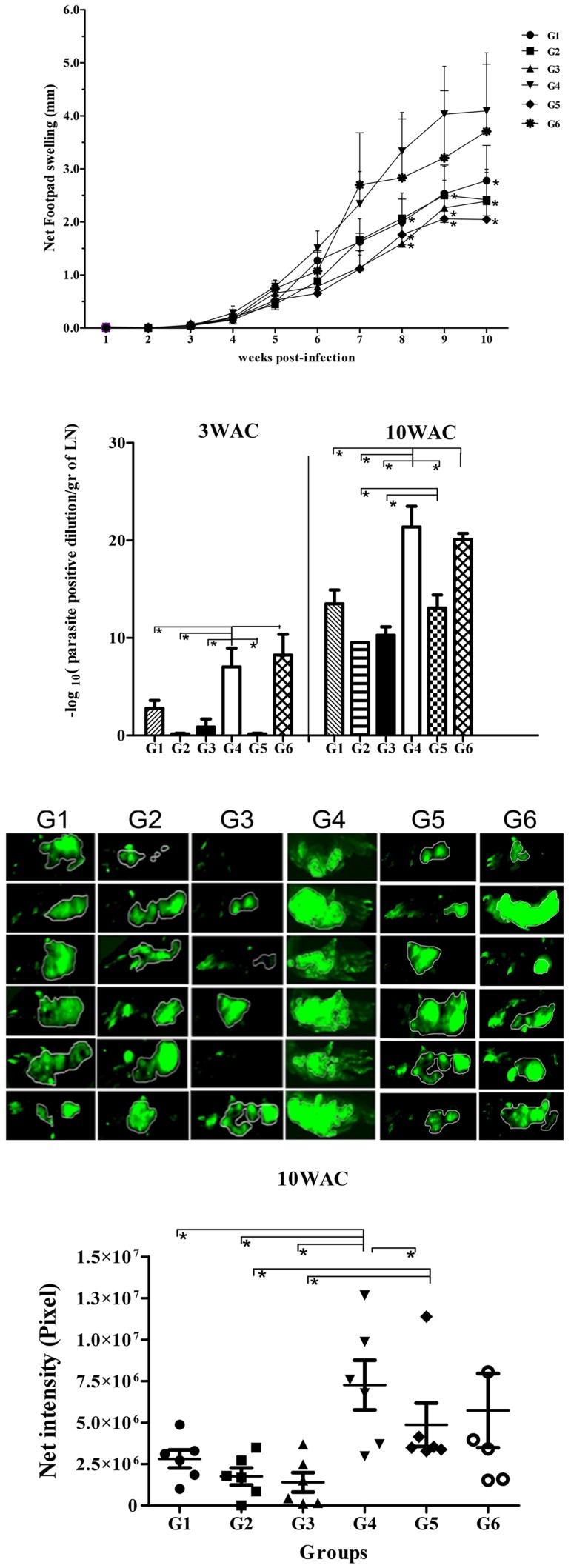
The course of infection with *L. major* GFP^+^ in BALB/c mice vaccinated with different modalities. BALB/c mice were immunized in the right footpad with *L. tarentolae CPA/CPB/EGFP* (G1, rLive/rLive); primed with DNA PpSP15 and boosted with *L. tarentolae CPA/CPB/EGFP* and DNA PpSP15 (G2, DNA/rLive+DNA); primed and boosted with both *L. tarentolae CPA/CPB/EGFP* and DNA PpSP15 (G3, DNA+rLive/DNA+rLive); injected with PBS as control (G4), vaccination and boosting with VR1020-SP15 (G5, DNA/DNA); G6: vaccination and boosting with *L. tarentolae* EGFP+ (G6, control Live/Live). All animals were challenged with stationary phase *L. major* (2×10^6^/mice) plus SGH (0.5 pair) in the left footpad except for G1 and G6, which received only *L. major*. A) The footpad swelling represents the Mean±SD of 12 mice per group; Asterisks indicate statistical significance (Mann Whitney U test, *p<0.05) compared to the control group (G4). B) Parasite burden per lymph node in all groups at 3 and 10 weeks post challenge (WAC). Each data point represents the Mean ±SD of 4 lymph nodes per group; statistics were carried out by ANOVA. C) Photographs of mouse footpads infected with fluorescent *L. major* for G1–G6. D) Net intensity by fluorescence imaging at 10WAC; statistical differences was determined by the Mann Whitney U test (*p*<0.05 denoted as *). Values of two independent experiments are shown in the figure.

All six groups of vaccinated and control BALB/c mice (Table 1) were challenged with 2×10^5^ late-stationary phase *L. major* GFP^+^ promastigotes in their left footpads in the presence (G2, G3, G4, G5) or absence (G1, G6) of SGH. Weekly measurements showed a sharp increase in footpad swelling in the control groups G4 and G6 at weeks 8, 9 and 10 that was significantly larger than that observed in groups G1, G2, G3 and G5 (Figure 4A *p*<0.05). As a main parameter, the parasite burden was measured in the lymph nodes of all six groups at 3 and 10 weeks post challenge using a limiting dilution assay (Figures 4B). Three weeks after challenge (3WAC), groups G1, G2, G3 and G5 showed a significantly lower parasite load than groups G4 and G6 (Figure 4B) with G2 and G5 showing the lowest lymph node parasite burden (Figure 4B). At the end of week 10, the parasite burden of groups G1, G2, G3 and G5 remained significantly lower (*p*<0.05) compared to groups G4 and G6 (Figure 4B). In addition, both G2 and G3 has significantly lower parasite load in respect to G1 and G5 (p<0.05). *In vivo* imaging of fluorescent parasites in the footpad 10 weeks after challenge (10WAC) shows a significant reduction in the level of fluorescence intensity in the footpad of the vaccinated groups G1, G2 and G3 as compared to the control group G4 (Figures 4C–D). Group G3 had the lowest fluorescence intensity with two mice showing no GFP fluorescence (Figure 4C–D). Moreover, the fluorescence intensity of group G3 was significantly lower in comparison to groups G1, G4, G5 and G6 (p<0.05) but was not statistically significant from that of group G2 (Figure 4D).

**Figure 5:**
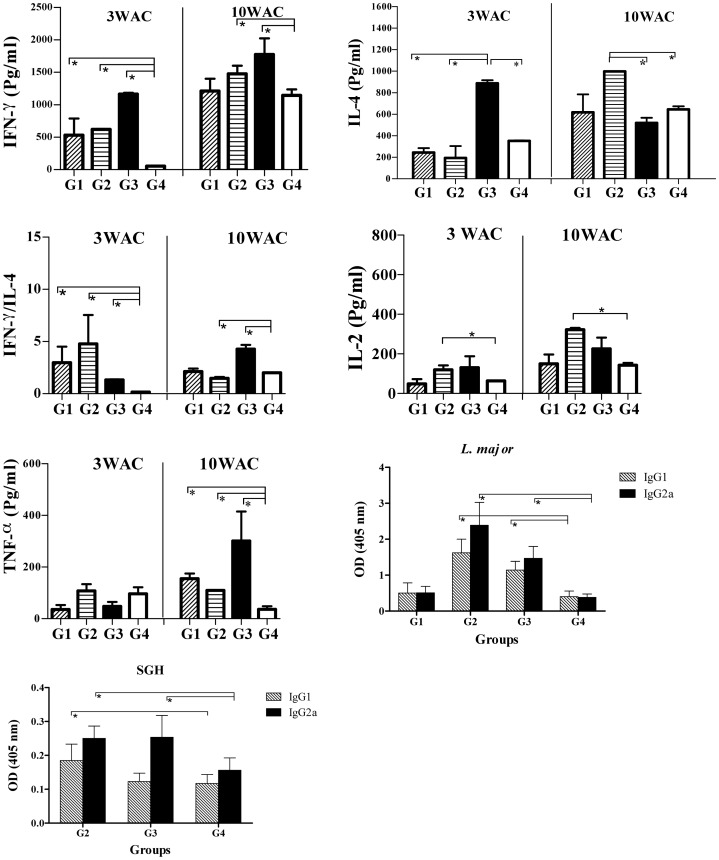
The immune response post-challenge in BALB/c mice vaccinated with different modalities. A–E, Cytokine production by splenocytes at 3 and 10 weeks after challenge (WAC). Single cell suspensions were prepared from splenocytes of 4 mice after infectious challenge. Cells were cultured in duplicate in the presence of freeze-thawed *L. major* +SGH for G2, G3 and G4 and with only *L. major* for G1. Culture supernatants were assayed for the level of IFN-γ (A), IL-4 (B), ratio of IFN-γ to IL-4 (C), IL-2 (D) and TNF-α (E) production by ELISA. Each bar represents the Mean±SD of 4 mice per group. The asterisk indicates the significant difference between values at the indicated time points as determined by Mann Whitney U test compared to the control group (G4). Antigen-specific antibody responses against *L. major* (F) and SGH (G) five weeks after challenge. Data represent the serum IgG1 and IgG2a levels of individual mice within their respective vaccination group (n = 8–10). Statistical analysis was carried out by Mann Whitney U test comparing vaccination groups against G4 as control (*p*<0.05 denoted as *). Results are representative of two independent experiments.

For assessment of the immune response after challenge, we focused on the groups vaccinated with *L. tarentolae* CPA/CPB/EGFP+ (G1) and a combination of *L. tarentolae CPA/CPB/EGFP+* and VR1020-SP15 (G2, and G3) compared to the PBS-immunized control group G4. Splenocytes stimulated with *L. major* F/T antigen at 3WAC show that groups G1, G2 and G3 produced significantly higher levels of IFN-γ compared to control group G4 (Figure 5A, *p*<0.05). Though group G3 produced higher levels of IFN-γ compared to group G2, it also produced significantly higher levels of IL-4 (*p*<0.05) as compared to groups G1, G2 and G4 (Figure 5B). The difference in the levels of these two cytokines became less pronounced at 10WAC (Figure 5B). All vaccinated groups showed a positive IFN-γ/IL-4 ratio and group G2 had the highest IFN-γ/IL-4 ratio at 3WAC indicative of a Th1 response (Figure 5C). At 10WAC, group G3 had the highest ratio of IFN-γ/IL-4 (*p*<0.05) in comparison to G1, G2, G4. With regard to IL-2 production, only G2 produced significantly higher levels of this cytokine as compared to G1, G3 and G4 at 3WAC and 10WAC (Figure 5D). TNF-α production was similar at in all vaccinated and control groups at 3WAC but it was significantly higher in the three vaccinated groups (G1, G2 and G3) compared to control group G4 at 10WAC (Figure 5E, *p*<0.05).

The specific antibody response against *L. major* in BALB/c mice was measured in the above-mentioned groups at 5 weeks after challenge. Groups G2 and G3 displayed the highest level of IgG2a and IgG1 antibodies to *Leishmania* compared to group G1, and control group G4 (p<0.05, Figure 5F). The low levels of IgG1 and IgG2a antibodies to *Leishmania* were similar in groups G1 and G4 (Figure 5F). Regarding anti-sand fly saliva antibodies, the levels of IgG2a antibodies were significantly higher in groups G2 and G3 compared to control group G4 (Figure 5G). Furthermore, the ratio of saliva-specific IgG2a/IgG1 was greater in groups G2 and G3 (Figure 5G).

### Evaluation of vaccine efficacy in resistant C57BL/6 mice

**Figure 6:**
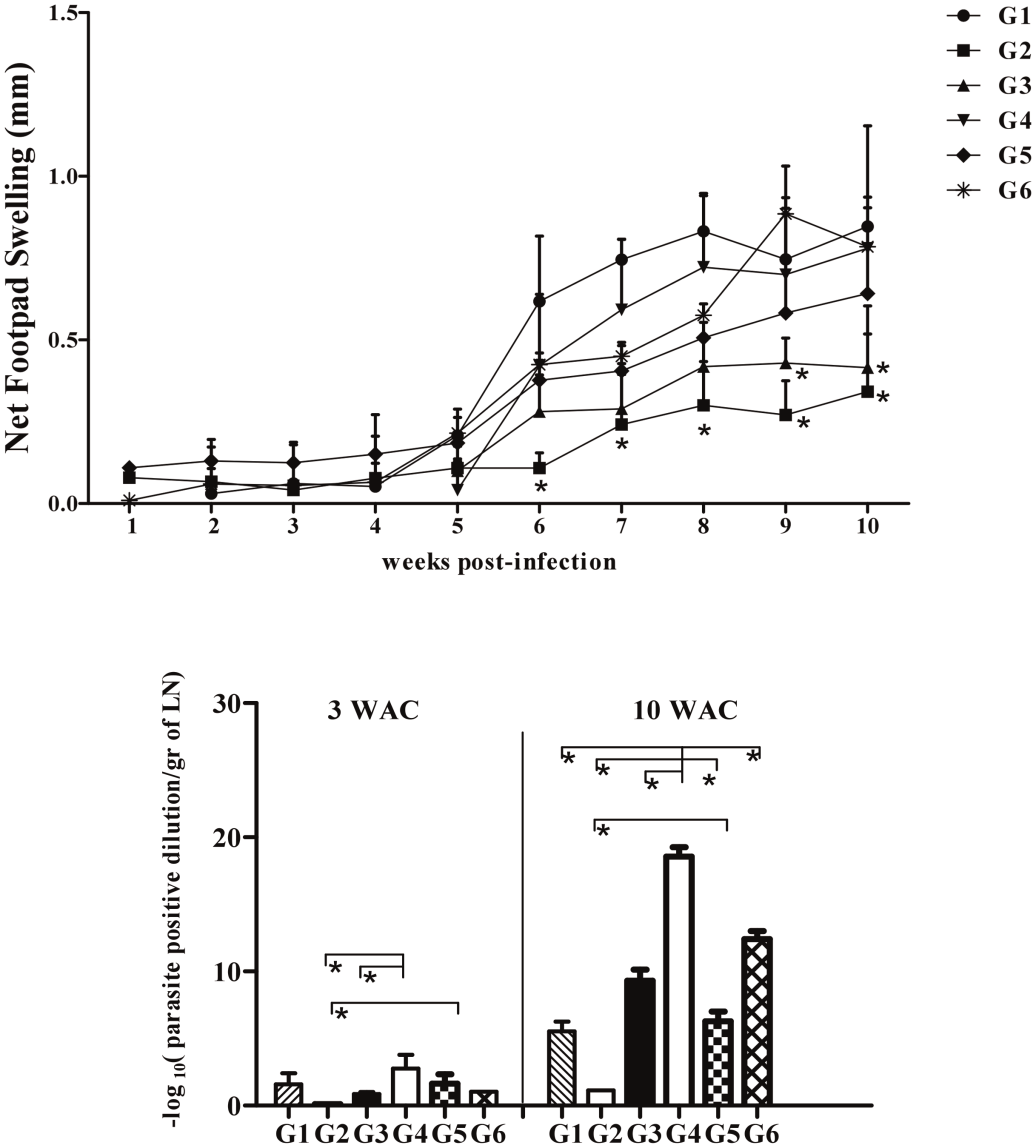
The course of infection with *L. major* GFP+ in C57BL/6 mice vaccinated with different modalities. C57BL/6 mice were immunized in the right footpad with *L. tarentolae CPA/CPB/EGFP* (G1; rLive/rLive); primed with DNA PpSP15 and boosted with *L. tarentolae CPA/CPB/EGFP* and DNA PpSP15 (G2; DNA/rLive+DNA); primed and boosted with *L. tarentolae CPA/CPB/EGFP* and DNA PpSP15 (G3; DNA+rLive/DNA+rLive); injected with PBS as control (G4), vaccination and boosting with VR1020-SP15 (G5, DNA/DNA); G6: vaccination and boosting with *L. tarentolae* EGFP+ (G6, control Live/Live). All animals were challenged with stationary phase *L. major* (2×10^6^/mice) plus SGH (0.5 pair) in the left footpad except for G1 and G6 which only received *L. major*. A) The footpad swelling represents the Mean±SD of 12 mice per group. Asterisks indicate statistical significance (Mann Whitney U test, *p*<0.05) compared to the control group (G4). B) Parasite burden per lymph node in all groups at 3 and 10 weeks post challenge (WAC). Each data point represents the Mean ±SD of 4 lymph nodes per group. Statistics were carried out by ANOVA (*p*<0.05 denoted as *). Values of two independent experiments are shown in the figure.

In C57BL/6 mice the increase in footpad swelling was similar between groups G1, G4 and G6 (Figure 6A). There was a significant decrease (*p*<0.05) in footpad swelling in groups G2 and G3 in comparison to groups G1, G4, G5 and G6 (Figure 6A). Measurements of parasite burden from lymph nodes at 3 and 10 weeks post-challenge showed that, group G2 had significantly the lowest parasite burden (*p*<0.05) as compared to groups G1, G3, G4, G5 and G6 (Figure 6B). We also observed at 10WAC a significant decrease in parasite burden in G1, G3 and G5 as compared to control group G4 as well as G6. In addition, the level of parasite burden in G6 is also significantly lower than G4 (*p*<0.05) (Figure 6B). Overall, these data demonstrate that in C57BL/6 mice priming with VR1020-SP15 and boosting with a combination of the live vaccine and VR1020-PpSP15 elicited a higher protective efficacy than the two other regimens in controlling footpad swelling and parasite propagation up to 10 weeks post-challenge (Figure 6A, B). Although we were able to detect the swelling in the footpad of all groups, we were unable however, to determine the fluorescent intensity by imaging in the footpad of C57BL/6 mice as it was done for BALB/c. In fact, in C57BL/6 mice resistant strain, the level of parasite propagation in the footpads of all groups was limited (below the instrument detection limit).

**Figure 7:**
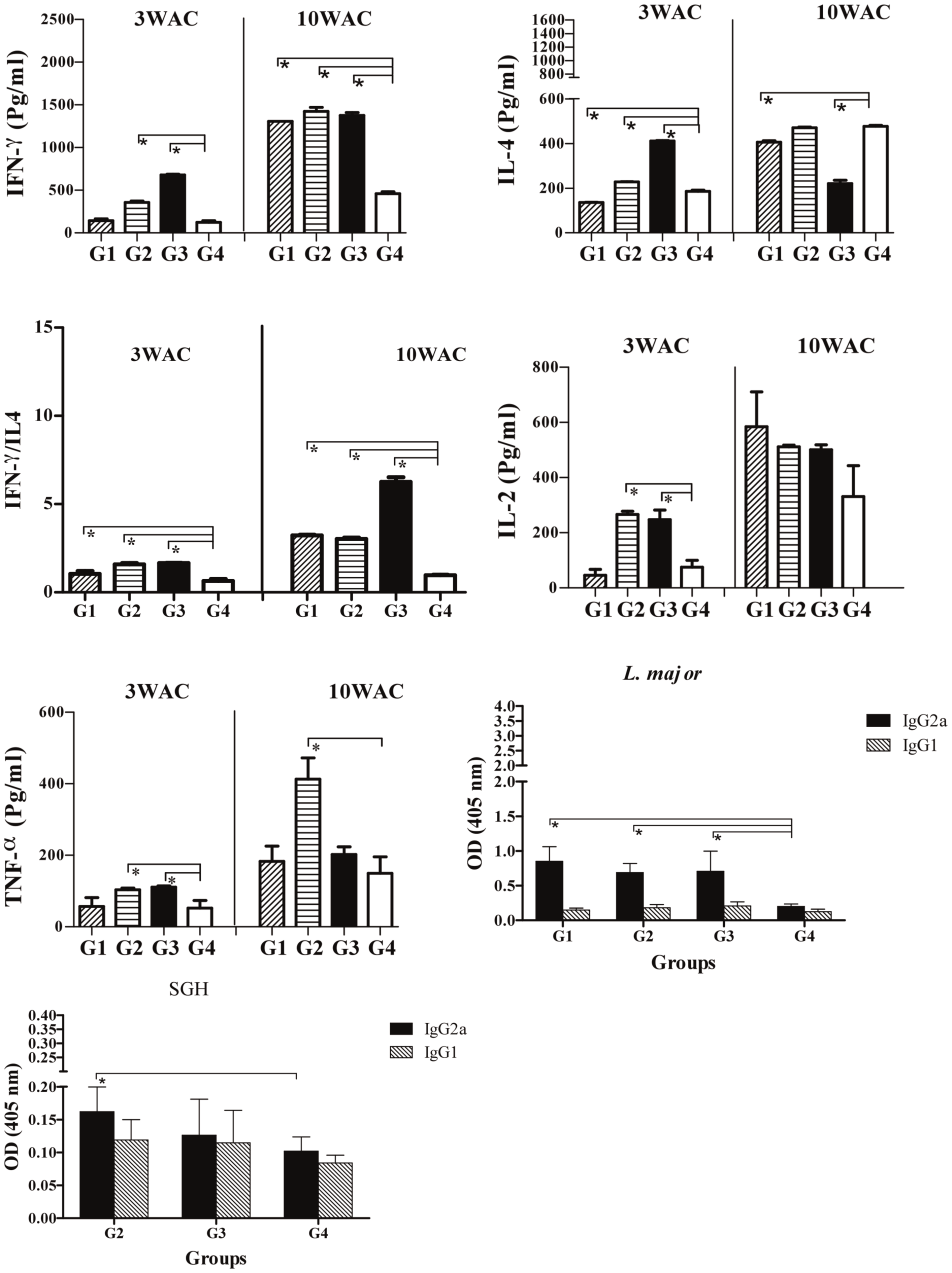
The immune response post-challenge in C57BL/6 mice vaccinated with different modalities. A–E, Cytokine production by splenocytes at 3 and 10 weeks after challenge. Single cell suspensions were prepared from splenocytes of four C57BL/6 mice at 3 and 10 weeks after infectious challenge. Cells were cultured in duplicate in the presence of freeze-thawed *L.major*+SGH for G2, G3 and G4 and with only *L. major* for G1. Culture supernatants were assayed for the level of IFN-γ (A), IL-4 (B), ratio of IFN-γ to IL-4 (C), IL-2 (D) and TNF-α (E) production by ELISA. Each bar represents the Mean±SD of 4 mice per group. Data were analyzed by the Mann Whitney test. Asterisks indicate the statistical differences between values at the indicated time points as compared to the control group (G4). Antigen-specific antibody responses against *L. major* (F) and SGH (G) five weeks after challenge. Data represent the serum IgG1 and IgG2a levels of each individual mouse within their respective vaccination group (n = 8–10). Statistical analysis was carried out by Mann Whitney U test (*p*<0.05 denoted as *). Results are representative of two independent experiments.

Similar to BALB/c mice, we focused on the groups vaccinated with *L. tarentolae* CPA/CPB/EGFP+ (G1) and a combination of *L. tarentolae CPA/CPB/EGFP+* and VR1020-SP15 (G2, and G3) compared to the PBS-immunized control group G4 for assessment of the immune response after challenge. Splenocytes stimulated with *L. major* F/T antigen at 3WAC show that groups G2 and G3 had higher levels of IFN-γ compared to control group G4 (Figure 7A). At 10WAC, IFN-γ production was similar among the vaccinated groups G1, G2 and G3 and was significantly higher than control group G4 (Figure 7A). The levels of IL-4 were lower in group G2 as compared to group G3 at 3WAC (Figure 7B, *p*<0.05), however this cytokine decreased significantly 10WAC in group G3 compared to groups G1, G2 and G4. Similar to BALB/c mice, the ratio of IFN-γ/IL-4 was higher in groups G1, G2 and G3 compared to the control group G4, particularly at 10WAC (Figure 7C). It is worth to mention that G3 has significantly the highest ratio in compare to G1 and G2 (*p*<0.05). As for IL-2, its production was significantly higher (*p*<0.05) in groups G2 and G3 compared to groups G1 and G4 at 3WAC, but no statistical significance was observed among the groups at 10WAC (Figure 7D). Importantly, the induction of TNF-α was significantly higher in Group G2 as compared to control group G4 at 10WAC and was 2-folds higher compared to G1 and G3 (Figure 7E).

Antibodies against *L. major* in vaccinated groups G1, G2 and G3 showed significantly higher levels of IgG2a in comparison to group G4 (*p*<0.05, Figure 7F). Furthermore, the levels of IgG1 were significantly lower than IgG2a in these three vaccinated groups (G1, G2 and G3) in comparison to G4 (*p*<0.05, Figure 7F). Overall, the antibody response to SGH was low (Figure 7G). With the exception of group G2 that produced significantly higher levels of IgG2a compared to groups G3 and G4, the antibody response to SGH was mixed (Figure 7G).

## Discussion

Despite substantial progress in fundamental *Leishmania* research, there are many unanswered questions concerning pathogenesis of the disease and the acquisition of protective immunity against reinfection. In this respect, immunization with live attenuated strains as a vaccine tool to induce a protective immune response in the host has a long tradition [20]. The major drawback of this approach is that under certain circumstances, the strains may gain virulence and become pathogenic again. To overcome this problem, subunit vaccines, instead of the whole organism, emerged as a vaccination strategy [21]. A number of parasite antigens have been tested for their potential to induce anti-*Leishmania* responses. The most extensively studied antigens using a wide range of adjuvants and delivery systems are GP63, LACK, CPs, and the poly-antigen Leish111f [22], [23], [24]. In an attempt to engage *Leishmania* infection at an early stage, salivary proteins of the sand fly have also been evaluated for vaccination. Studies in mice, hamsters and dogs showed promising results with the induction of Th1-like responses and long-term protection against both cutaneous and visceral infections using these salivary proteins [4], [8].

Here, we describe the outcome of a new vaccination strategy with different modalities using a live recombinant nonpathogenic *L. tarentolae* vaccine expressing *CPA/CPB/EGFP* combined to a DNA vaccine containing the cDNA for PpSP15, the predominant 15 kDa salivary protein from the sand fly *P. papatasi*. Our target parasite antigens are cysteine proteinases, which are conserved among different *Leishmania* species and are highly immunogenic. *L. tarentolae*, the lizard protozoan parasite, has been previously introduced by Breton *et al.* [25] as a candidate vaccine against visceral leishmaniasis. Furthermore, we have demonstrated that a recombinant *L. tarentolae* strain expressing the *L. donovani A2* gene elicited a strong protective immunity against virulent *L. infantum* challenge [26]. Recently, we have shown that vaccination with *L. tarentolae* expressing *A2/CPA/CPB* induced a strong parasite-specific Th1 response and conferred protection against *L. infantum* challenge in BALB/c mice [11]. As for PpSP15, it was shown previously to protect vaccinated C57BL/6 mice challenged with parasites plus SGH [15], [27].

A major requirement of vaccines in general, is that they are able to protect the majority of a population, which normally displays a high diversity in MHC haplotypes. For this reason, we tested the efficacy of the recombinant live *L. tarentolae* expressing *CPA/CPB/EGFP* candidate vaccine combined to PpSP15 DNA in eliciting protective immune responses in two different strains of mice. While BALB/c mice develop progressive lesions upon infection with *L. major*, C57BL/6 mice are naturally resistant and the infection normally causes transient symptoms (contained lesion development and visceralization) and is self-healing. In this study, *L. major* IR75 was used for an infectious challenge because it is more virulent in comparison to *L. major* 39 and the Friedlin strain (Modabber F, personal communication). Both strains of mice showed the strongest protective effect following immunization with a prime/boost regimen based on PpSP15 DNA and recombinant *L. tarentolae* (groups G2 and G3) demonstrating an enhanced vaccine efficacy compared to the sole use of *L. tarentolae CPA/CPB/EGFP* (G1) or PpSP15 DNA (G5). While group G3 showed a more potent immune response in susceptible BALB/c mice, group G2 showed the strongest immunogenicity in C57BL/6 mice and it was the best group in controlling parasite growth in the lymph nodes of both mice strains. In both strains of mice, immunization with PpSP15 as a DNA vaccine combined to *L. tarentolae CPA/CPB/EGFP* showed considerable level of protection as demonstrated by footpad thickness measurements and parasite burden. This demonstrated for the first time the effectiveness of co-immunization of a sand fly salivary protein, PpSP15, with live *L. tarentolae CPA/CPB/EGFP* in controlling the disease. In the case of BALB/c mice, the effect of Live *L. tarentolae CPA/CPB/EGFP* is less pronounced although we observed a significantly lower parasite burden in G2 and G3 compared to G1, G4, G5 and G6. Inclusion of PpSP15 DNA as a vaccine may be relevant at two levels: i) as an inducer of adaptive immunity, thus reducing lesion pathology and parasite propagation and ii) as an potential enhancer of innate immunity due to the intrinsic properties of this molecule that may contribute to the control of intracellular growth of *L. major*. Furthermore, there are extensive data showing that live *L. major* plus CpG DNA prevents lesion development and causes the specific induction of Th17 cells, which enhances the development of a protective cellular immunity against the parasite [28], [29]. Data presented by Mendez *et al.* [30] suggest that a vaccine combining live pathogens with immunomodulatory molecules may strikingly modify the natural immune response to infection in an alternative manner to that induced by killed or subunit vaccines. Therefore, it may be possible that PpSP15 working as an immunomodulatory molecule and enhancing the development of a protective cellular immunity against the parasite.

Comparing the data obtained in C57BL/6 with BALB/c, the highest level of TNF-α production, indicative of a Th1 response, was seen with group G2 at 10WAC although there were no significant differences in IFN-γ production. Of note, we only checked four key cytokines to demonstrate the immunogenicity of each vaccine modality using the live recombinant *L. tarentolae*. We acknowledge the need to further investigate the role or contribution of other cytokines when studying live parasite vaccines. Our future efforts should be also focused on the analysis of the immunological memory and the factors that could correlate with the size of the memory pool using these vaccine strategies. One of these aspects is the induction of CD8 T+ cell responses, which remains to be elucidated.

The concept of using live vaccination against leishmaniasis is not new. Actually, the inoculation of live parasites to produce a lesion that heals, named leishmanization, has been the only vaccination strategy implemented at a large scale because it provides lifelong protection against the development of additional lesions [31]. However, this approach was discontinued because of raised non-healing or slow healing lesions in several human cases [31], [32]. During the last few years, several attenuated strains of *Leishmania* have been developed. As an alternative, various defined genetically modified parasites have been achieved using a gene targeted disruption strategy through homologous recombination. One of the first examples was the *in vivo* evaluation showed that the *dhfr-ts−/−*parasites survived but were unable to establish a persistent infection or to cause disease even in the most susceptible mouse strains [33]. Other examples such as LPG2−/− parasites protected highly susceptible BALB/c mice against a *L. major* virulent challenge even in the absence of a strong Th1 response [34], [35]. In contrast to *L. major* mutants, *L. mexicana* LPG2−/− mutants retained their virulence for macrophages and mice [35], which suggested that different *Leishmania* species possess alternative virulence repertoires to interact with their host. Therefore, major safety constrains, such as a possible reversion to virulence or reactivation in immunosuppressed individuals, are still among the limiting factors against the use of such vaccines. In contrast to all of the above-mentioned approaches, *L. tarentolae* is non-pathogenic to humans and can be used in immunocompromised individuals. As such, recombinant *L. tarentolae* could offer more advantages for vaccine development not only against *Leishmania*, but also against other pathogens. A recombinant *L. tarentolae* expressing HIV-1 Gag protein induced strong cell-mediated immunity in mice and decreased HIV-1 replication in an *ex vivo* system, suggesting that this species can be applied as a promising live vaccine against intracellular pathogens [10]. Recently, a recombinant *L. tarentolae* strain expressing HPV-E7 antigen-green fluorescent protein (GFP) was developed and showed a potential as a live vaccine against HPV infection [36]. Additionally, modification of, and insertion into, the genome of *L. tarentolae* can be done easily and there is no insert size limitation making it a versatile tool for vaccine development.

Our data clearly demonstrate that group G2 (prime with PpSP15 DNA and boost with *L. tarentolae CPA/CPB/EGFP*+PpSp15 DNA) has the lowest level of parasite propagation at 3WAC in both mice strains and at 10WAC in C57BL/6 mice. Therefore, apart from the specific immunogenicity of PpSP15, this salivary protein may have an immunomodulatory role that in combination with a live vaccine potentially enhances its efficacy against *Leishmania*.

In summary, the present study suggests that this new approach that combines a prime-boosting strategy using recombinant *L. tarentolae* with a sand fly salivary protein offers a promising platform for developing a more effective vaccine against leishmaniasis.
