# Expression of Concern: Enhanced Protective Efficacy of Nonpathogenic Recombinant *Leishmania tarentolae* Expressing Cysteine Proteinases Combined with a Sand Fly Salivary Antigen

**DOI:** 10.1371/journal.pntd.0009123

**Published:** 2021-02-17

**Authors:** The PLOS Publication Ethics Editors

Following publication of this article [1], concerns were raised about duplication of image panels in Fig 4C. Due to an error in assembly of the originally published Fig 4C, image panels in Group G3 rows 2, 3, 4 and 6 are incorrect, showing images from the same samples as Group G5 rows 1, 2, 3, and 5, respectively. A revised Fig 4 is provided using the correct Group G3 images and a replacement Group G5 row 2 panel, all from the original experiment.

**Fig 4:**
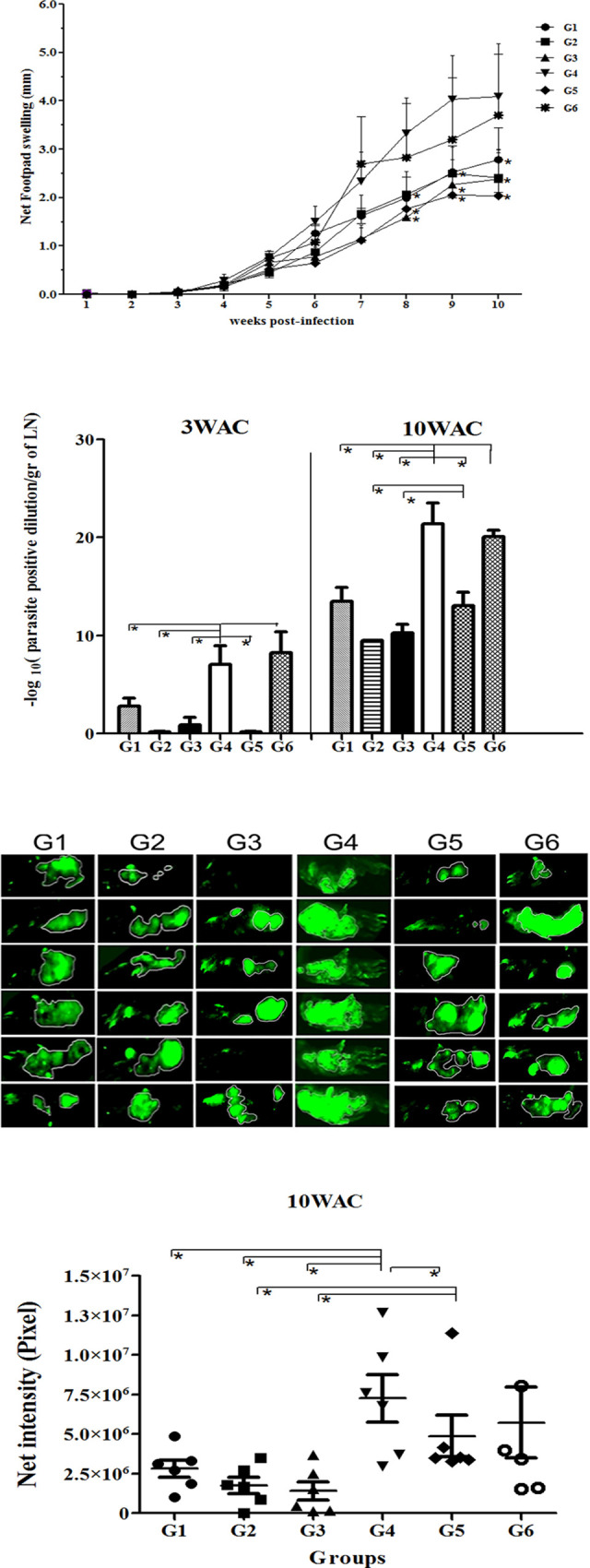
The course of infection with L. major GFP+ in BALB/c mice vaccinated with different modalities. BALB/c mice were immunized in the right footpad with *L. tarentolae CPA/CPB/EGFP* (G1, rLive/rLive); primed with DNA PpSP15 and boosted with *L. tarentolae CPA/CPB/EGFP* and DNA PpSP15 (G2, DNA/rLive+DNA); primed and boosted with both *L. tarentolae CPA/CPB/EGFP* and DNA PpSP15 (G3, DNA+rLive/DNA+rLive); injected with PBS as control (G4), vaccination and boosting with VR1020-SP15 (G5, DNA/DNA); G6: vaccination and boosting with *L. tarentolae* EGFP+ (G6, control Live/Live). All animals were challenged with stationary phase *L. major* (2×10^6^/mice) plus SGH (0.5 pair) in the left footpad except for G1 and G6, which received only *L. major*. A) The footpad swelling represents the Mean±SD of 12 mice per group; Asterisks indicate statistical significance (Mann Whitney U test, *p<0.05) compared to the control group (G4). B) Parasite burden per lymph node in all groups at 3 and 10 weeks post challenge (WAC). Each data point represents the Mean ±SD of 4 lymph nodes per group; statistics were carried out by ANOVA. C) Photographs of mouse footpads infected with fluorescent *L. major* for G1–G6. D) Net intensity by fluorescence imaging at 10WAC; statistical differences was determined by the Mann Whitney U test (*p*<0.05 denoted as *). Values of two independent experiments are shown in the figure.

In addition, concerns were raised that in the originally published figure, the images in Group G3, row 3 (G3M3), and Group G5, row 2 (G5M2), are highly similar, but the two images differ in fluorescence intensity of the marked heel region on the right-hand side of the image panel.

Upon editorial follow-up, the authors provided two different versions of underlying image files for the G5M2 sample (A and B versions, S1 File), which differ in fluorescence intensity of the heel region on the right-hand side of the image. The authors commented that differing fluorescence may be explained by the application of normalization procedures to reduce background autofluorescence (please see S2 File for a description of image acquisition and analysis methodology); image analysis parameters were adjusted using a normal mouse footpad as reference, and this normalization was applied to all images in the experiment. The dimmer “B” version of the G5M2 image is reported by the authors to be a pre-normalization image saved erroneously, while the brighter “A” version is considered consistent with the image after normalization is applied. Neither of the two underlying images are identical to the image used in the G5M2 panel of the originally published Fig 4C, and it has not been possible to reproduce the identical image because the original instrument is unavailable and the precise settings used are not known. In the revised Fig 4, the G5M2 panel has been replaced using the G5M2 “A” underlying image.

Review of the underlying data was carried out in consultation with an independent expert who advised that to fully clarify this issue we would require the original .BIP files captured at the time of image analysis. However, the raw .BIP files and the full information about parameter settings used at the time of the original analysis are not available. In the absence of the raw data, it has not been possible to fully clarify the reason(s) underlying the differences between the images of the G5M2 footpad, and we have been unable to verify how the images reported in the figure align with the original experimental output.

The authors stated that all intensity data used for the chart and statistical analysis in Fig 4D are derived from the raw quantitative data output of the imaging instrument and are unaffected by any adjustments to images in Fig 4C. The available quantitative data (S3 File) and processed image files (TIFFs, S4 File) for Fig 4 are provided here as Supporting Information. Individual-level data underlying the chart in Fig 4A are unavailable. All other underlying data for the article are available from the corresponding author upon request.

In light of the concerns about the reporting of image data in Fig 4C, which could not be fully clarified because the raw image data are not available, the PLOS Publication Ethics Editors issue this Expression of Concern.

## Supporting information

S1 File Fig 4C underlying images G5M2. (ZIP)

S2 File Image acquisition and analysis methodology. (DOCX)

S3 File Fig 4 Chart Data. (ZIP)

S4 File Fig 4C underlying TIF images. (ZIP)
