# Supplementary figures and images for: Expression of Concern: Enhanced Protective Efficacy of Nonpathogenic Recombinant Leishmania tarentolae Expressing Cysteine Proteinases Combined with a Sand Fly Salivary Antigen
Source: PLoS Negl Trop Dis. 2021 Feb 17;15(2):e0009123. doi: 10.1371/journal.pntd.0009123 (PMC7888667; doi:10.1371/journal.pntd.0009123)

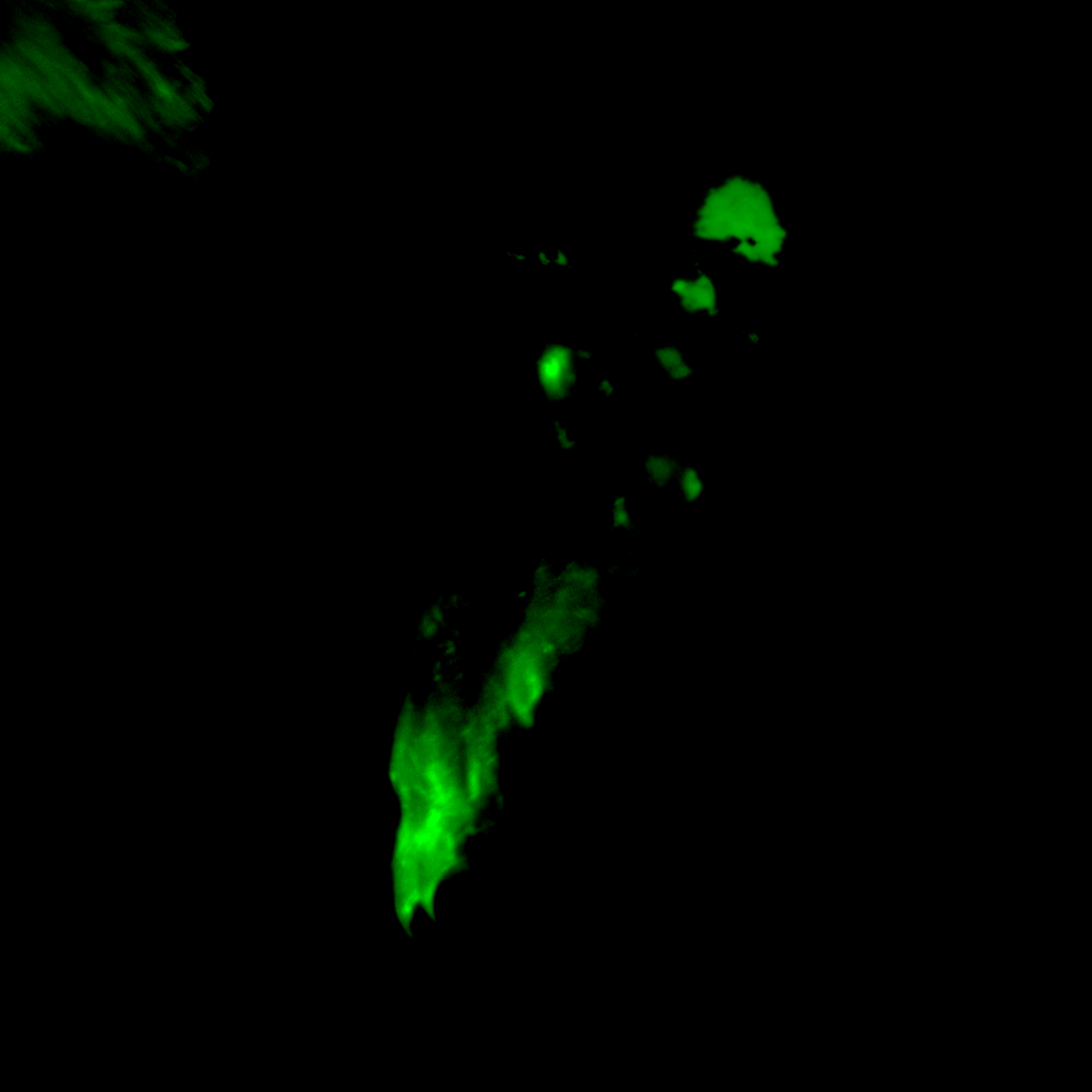

Supplement: S1 File — (ZIP) [file pntd.0009123.s001.zip › G5-M2 A.tif]
