# Supplementary material for: Expression of Concern: Enhanced Protective Efficacy of Nonpathogenic Recombinant Leishmania tarentolae Expressing Cysteine Proteinases Combined with a Sand Fly Salivary Antigen
Source: PLoS Negl Trop Dis. 2021 Feb 17;15(2):e0009123. doi: 10.1371/journal.pntd.0009123 (PMC7888667; doi:10.1371/journal.pntd.0009123)
Supplement: S2 File — (DOCX) [file pntd.0009123.s002.docx]

The detailed description of the procedure used at the time is as follows:

1. Mice were temporarily anesthetized intraperitoneally with a mixture of xylazine 2% (7.5 ul), Ketamine 10% (30ul) and saline solution (260ul) per mouse.
2. Mice were individually positioned on the imaging stage.
3. A few mice were randomly selected for adjusting the instrument’s settings including the f-stop, FOV, and GFP mode to optimize exposure time and filter wavelength (excitation/emission). The optimized parameters are as follows: f-stop=2.51; FOV=22.1mm; GFP=470 (Excitation) and 535 (emission); exposure time= 5.0 secs. These optimized parameters were then applied to the rest of the imaging procedure.
4. To reach the best-normalized capture mode, other parameters including “Min (in green), Gamma (in red) and Max (in blue) were adjusted and the optimum number for each parameter was fixed and applied to all images. To minimize error, setup images used for normalization were not stored, and only final images, captured after optimization of the above parameters, were saved.
5. After each single mouse was imaged, the captured image was first saved as *.bip and then as *.tif files.
6. For analysis, pixel counting and measurement of the lesions were performed using the KODAK molecular image software version 5.3 on the instrument. To this end, the target regions (regions of interest) were gated and the fluorescent intensities were quantified as “Net intensity” (a quantitative measurement defined as the number of green pixels in a given area multiplied by the average intensity of each pixel minus the background intensity) and saved/exported as an excel file.

The *.tif files were used for figure illustrations while the *.xls files were used for statistical analysis. The *.xls files denote values of target regions obtained directly from the machine readout and were used for statistical analysis of the difference in fluorescence intensity between groups.

1. To prepare Figure 4C, the target regions were estimated by lines and cropped images were lined up together accordingly in one single panel.

**Note:** Since the instrument was not accessible out of the Biotechnology department, we could not transfer the *.bip files to our lab at the time.
